# A Physical Inquiry into the Powers and Operation of Medicines

**Published:** 1790

**Authors:** Thomas Percival


### IX. *A physical Inquiry into the Powers and Operation of Medicines. By* Thomas Percival, *M. D. F. R. S. and S. A. Lond. F. R. S. and R. M. S. Edinb. &c. — From the Memoirs of the Literary and Philosophical Society at Manchester, Volume the Third.*

Causa latet, vis est notissima.

OVID.

*To the Literary and Philosophical Society.*

Manchester, Nov. 25, 1786.

MEDICINES are the instruments employed for the preservation of health, or the cure of diseases: it must, therefore, be an object of interesting speculation to the philosopher, and of practical importance to the physician, to investigate the rationale of their action on the human body. But there is no branch of the healing art which is in itself more intricate and obscure; nor any one that has undergone so many doctrinal vicissitudes, It would trespass too much on the time allotted for such discussions, in this Society, to enumerate the multifarious hypotheses which have been been ſupported by the ſucceſſive ſectaries in phyſic ſince the days of Hippocrates. On many of theſe I have animadverted in a work publiſhed near twenty years ago*: and I ſhall now only requeſt your candid attention to a few obſervations on the opinions which are beginning to prevail in our ſchools; and your permiſſion to offer ſome hints towards the extenſion of our views, and the methodiſing of our experience, relative to this curious and philoſophical ſubject.

Anatomy has now revealed the exquiſite ſtructure of our corporeal frame; and phyſiology has taught us that, in its animated ſtate, the organs of which it is compoſed are reciprocally connected with, and delicately adjuſted to, each other. The minuteſt agent, therefore, may excite a movement capable of being propagated to any part of the ſyſtem, or even through the whole of it, by a ſympathetic energy, independent and far beyond the power of the primary inſtrument of motion. From theſe premiſes it is inferred, agreeably to the ſimplicity which ſubſiſts in all the operations of nature, that a medicine is only the *cauſe of a*

* Eſſays Medical and Experimental, Vol. I.

*cauſe,*

*cause*, to adopt a phrase of the logicians; and that its proper action is confined to the nerves or fibres to which it is immediately applied. When received into the stomach, after the first impression on the very sensible coats of that organ, the nature of it is gradually changed by the solvent powers of the gastric juices; or, if incapable of being digested into a mild and nutritious chyle, it is carried through the intestinal canal, and ejected as useless and noxious to the body.

Error may be built on the basis of acknowledged, if only partial, truth, and is then most specious in its form, and most authoritative in its influence on the understanding; but the imposition ceases when we extend our views: and I shall endeavour to shew that the operation of medicines is to be measured by a more enlarged scale than the foregoing hypothesis applies to it, or any other which now occurs to my recollection.

I. Medicines may act on the human body by an immediate and peculiar impression on the stomach and bowels, either in their proper form; in a state of decomposition; or by new powers acquired from combination, or a change in the arrangement of their parts. The sympathy pathy of the ſtomach with the whole animated ſyſtem is ſo obvious to our daily experience, that it cannot require much illuſtration. After faſting and fatigue, we feel that a moderate quantity of wine inſtantly exhilarates the ſpirits, and gives energy to all the muſcular fibres of the body. It has been known even to produce a ſudden and large augmentation of weight, after much depletion, by rouſing the abſorbent ſyſtem to vigorous action. Such power is peculiar to living mechaniſm, and is properly denominated by phyſicians the *Vis medicatrix naturæ*. But apparent as is the ſympathy of the ſtomach, the laws by which it is governed are very inſufficiently underſtood; and we have hitherto learned only from a looſe induction of facts, that the nerves of this delicate organ ſeem to be endued with diverſified ſenſibilities; that impreſſions, made by the ſame or different ſubſtances, have their appropriate influence on different and diſtant parts; and that the ſtomach itſelf undergoes frequent variations in its ſtates of irritability. A few grains of blue vitriol, taken internally, excite inſtantly the moſt violent contractions in the abdominal and other muſcles concerned in vomiting. A doſe of ipecacuanha, as ſoon as it produces nauſea, naufea, abates both the force and velocity of the heart in its vital motions, and affects the whole feries of blood veffels from their origin to their minuteft ramifications, as is evident by the palenefs of the fkin under fuch circumftances, and by the efficacy of emetics in ftopping hæmorrhages. The head, when diforder-ed with vertigo, fometimes derives fudden relief from a tea-fpoonful or two of æther, adminiftered in a glafs of water: and I have known an inceffant cough to attack the lungs in confequence of the ftimulus of a pin which had been unwarily fwallowed. Of the action of medicines on the ftomach, under decompofition or recompofition, we have an example, familiar to every one, in magnefia; for this abforbent earth, by neutralifing the acid in the primæ viæ, acquires a purgative quality, and at the fame time yields a gas of great falubrity, as an anti-emetic, tonic, and antifeptic.

II. Medicines may pafs into the courfe of circulation in one or other of the ftates above defcribed; and, being conveyed to different and diftant parts, may exert certain appropriate energies. Chemiftry furnifhes numberlefs cafes wherein fubftances undergo changes, and put on new forms more remarkable than can be effected by the digeftion of the ftomach, retaining ftill the *materia prima*, and being capable of refuming the original arrangement of their particles, and confequently their original qualities. Now, a body altered in its texture by the digeftive organs, and carried into the fyftem with the aliment, may, by fuch alteration, acquire fpecific powers on particular found or difeafed parts. Thus if we fuppofe cantharides to be changed in form and texture, when mixed with the chyle, the lymph, or blood, yet in that form and texture they may be peculiarly adapted to excite ftrangury in the urinary paffages; or we may conceive that this new modification of their corpufcles may again be altered, and their original compofition reftored by a fubfequent chemical change in the kidnies — an event not more fingular than the feparation of urine from the blood, than the revival of a metal, or the precipitation of a folvend from its menftruum by elective attraction. The urinary excretion feems to be defigned by nature to carry off the recrementitious parts of the circulating fluids: and it clearly fhews what compofitions and decompofitions take place in the body; for it varies almoft every hour both in the ftate of health and of difeafe, and the lateritious, ritious, pinky, mucous, and other appearances it exhibits, are the refult of chemical changes, either in itfelf, or in the fluids from which it is derived.

The fenfible qualities of any body are no certain criteria of its medicinal action. Peruvian bark owes not its efficacy to bitternefs; for ftronger bitters are not poffeffed of its febrifuge powers. Antimony, though infipid, is violent in its operation on the nerves of the ftomach; and yet, if applied to the eye, an organ endued with equal fenfibility, it is entirely inert. To what property in opium, capable of affecting the external fenfes, are we to afcribe its narcotic powers? or is there in the grateful tafte of faccharum faturni any indication of a deadly poifon? But the inftances are numberlefs which may be adduced to prove the uncertainty of reafoning, otherwife than from obfervation, concerning the action of medicines, and the peculiar fenfibility of different parts of our fyftem to their impreffion. Following, therefore, experience as our guide, let us notice fuch facts as may elucidate the fubject before us. It is well known that madder root carries its tinging quality to the bones, affecting neither the fkin, the mufcles, the ligaments, nor fat. Digeftion confequently confequently leaves this tinging quality unchanged; or perhaps it is again recovered, when arrived at the bones, by fome new arrangement of parts produced by the chemiftry of nature. Extract of logwood, taken internally, fometimes gives a bloody hue to the urine; but the aftringency of it does not, according to my trials, accompany its colouring matter *. I recollect no inftance wherein the milk either of a nurfe, or of an animal, was tinged with madder or logwood. This affords fome prefumption that the pigment does not fubfift, in its proper form, in the blood; but that it is recovered by a fubfequent change in the difpofition of its conftituent particles: and if one fubftance ftain the

* It is faid the fruit of the Nopal, or Indian fig, on which the cochineal is propagated, tinges the urine of thofe who eat it with a deep blood colour. The leaves of this fhrub are of a permanent and lively green; and it is remarkable that their juices are converted, by the concoctive organs of the infect which feeds on them, into a dye exactly fimilar to that produced by the powers of vegetation in the pulp of the fruit. Cartheufer obtained from the cochineal a moderately aftringent fpirituous extract, amounting, in weight, nearly to three fourths of the fubftance from which it was prepared. Thefe facts exhibit a ftriking analogy between digeftion and vegetation, as the products appear to be the fame both in colour and quality.

bones, bones, by being carried into contact with them, another may, in a way analogous, produce in them fragility or diffolution. In the difeafe termed, by the French, *ergot*, and which, with apparent reafon, is afcribed to the ufe of a fpecies of unfound corn, the bones lofe the earthy matter that enters into their texture, and become foft and eafy to be broken. This effect is gradual, and probably arifes from fome unknown quality of the corn, which is either not fubdued by digeftion, or refumed in the juices that circulate through the offeous veffels. A change in the procefs of vegetation may communicate a diffolvent power to an efculent feed. Muftard acquires it by its natural growth, and is capable of rendering even ivory itfelf foft and fragile. How far it would produce fuch an effect on the bones of a living body, if ufed as the chief article of diet, we have no experience on which to ground any fatisfactory conclufion. The peafantry, in the mountainous part of this country, who live on oatmeal, are peculiarly liable to the itch, and to other cutaneous eruptions. Thefe have fometimes been afcribed to obftructed perfpiration; but fuch obftruction is itfelf only a concomitant effect of fome quality in the oatmeal injurious to the fkin.

Sulphur,

Sulphur, whether externally or internally ufed, produces a cure in the itch. In each way, therefore, we may prefume its operation to be fimilar. But when taken into the ftomach there can be no doubt that it undergoes a change in the modification of its parts, and that it does not circulate through the blood veffels either in the form, or with the properties of fulphur; yet when conveyed to the furface of the body it appears evidently to recover its original powers, communicating its peculiar odour to the perfpiration, tinging filver, and curing cutaneous defœdations *. The fame holds true of the vitriolic acid, when adminiftered in large dofes. It feems to acquire phlogifton in the animal body, and to pafs off by the pores, as hepatic air, or as volatilifed fulphur. Even when given to nurfes, it proves an effectual remedy for the itch both in them and the children whom they fuckle. Mercury, combined

* Bifhop Watfon, in his Chemical Effays, warns thofe who ufe cofmetic lotions, containing ceruffe, to forbear from them at Harrowgate, Moffat, or other places where they drink fulphurated waters, " left they fhould be in the ftate of " the unlucky fair one, whofe face, neck, and arms, were " fuddenly defpoiled of all their beauties, and changed quite " black." Vol. III. p. 365.

with with ſulphur into an æthiops, has been generally regarded as inert: but inſtances have occurred in which, under this form, though accurately prepared, it has produced ſalivation—an evident proof of a chemical change in the æthiops, by which the mercury was reſtored to its priſtine powers. Indeed the ſame reaſoning may be applied to the ſpecific action of mercury on the ſalival glands, in whatever mode it be adminiſtered *. A ptyaliſm is ſometimes produced by antimony. Dr. James aſſured my friend, Sir George Baker, that he knew ſix inſtances of it occaſioned by his fever powder, although he had left mercury out of its compoſition long before they occurred; but the patients thus affected had neither their teeth looſened nor their breath made offenſive.

Moſt perſons have experienced the effects of aſparagus on the urine †. This takes place very

* We have the concurrent teſtimony of many authors, that mercury has been found, reſtored to its original form, in the carious bones of their patients.—Vide Joan. Fernel. cap. 7; Gabriel. Fallop. cap. 78; Joan. Lang. lib. i. epiſt. 43; Alex. Petron. cap. 1. lib. vi. &c. &c.

† Cabbage, eſpecially that of the winter's growth, impregnates water with a diſagreeable ſmell, ſomewhat ſimilar to very ſpeedily, and ſtrongly too, even though a ſmall quantity only has been eaten. The ſmell is much more diſagreeable than that of aſparagus itſelf: and as the odorous particles conveyed to the kidnies muſt be greatly diluted in their paſſage, (even on the ſuppoſition of the retrograde motion of the lymphatics, which does not ſeem probable in a caſe ſo invariable and uniform) I ſhould conceive that a new combination of particles takes place in the urinary organs, and that the odorous part of the ſecretion differs in its form and quality, both from what ſubſiſted in the chyle and in the blood.

There are certain medicines which, when ſwallowed, quickly manifeſt themſelves in the diſcharges with ſome of their original qualities. Soap lees, when taken in large quantities, render the urine alkaline and lithonthriptic; and the ſame excretion becomes impregnated with fixed air, if mephitic water be drank freely. A patient, whom I viſit at this time, has ſix grains of the balſam of tolu adminiſtered to him thrice every day, and his urine is ſtrongly to that which is communicated by aſparagus: yet, I believe, cabbage is never known to taint the urine; perhaps from its having no chemical affinity with it.

ſcented

ſcented even by this ſmall quantity. Fuller aſſerts of the balſam of copaiba, *Urinam odore violaceo minime inficit; illam vero ſapore amaro imbuit.* Garlick affects the breath, though applied only around the wriſts. The milk of a nurſe is likewiſe eaſily tainted with it. A purgative given to one who ſuckles will ſometimes produce no operation on her bowels, if ſhe be coſtive, but a powerful one on the child at her breaſt. But a ſtill more convincing proof that there may be a renovation of the original qualities of a body, after it has undergone the proceſs of digeſtion and other ſubſequent changes, is deducible from theſe facts — that butter is often impregnated with the taſte and ſmell of certain vegetables on which the cows have paſtured; that the milk of ſuch cows diſcovers no diſagreeable flavour; neither does the whey nor cheeſe prepared from it. Now butter is formed firſt by a ſpontaneous ſeparation of cream; and ſecondly by a fermentation of it, that is, by a two-fold and ſucceſſive new arrangement of its elementary parts. By theſe changes the original offenſive materials in the food of the cow ſeem to reaſſume their proper form and nature.

After venæſection the ſerum of the blood has ſometimes appeared as white as milk, whilſt

 the the craſſamentum retained its natural colour. This whiteneſs hath been ſhewn to ariſe from oleaginous particles (not unaſſimilated chyle) floating in the circulating fluids *; and may ſerve to explain a fact, recorded by a writer of good authority, on the natural hiſtory of Aleppo, that "in certain ſeaſons, when oil is plentifully taken, the people become diſpoſed to "fevers and infarctions of the lungs; which "ſymptoms wear off by retrenching this in-"dulgence †." Some years ago cod-liver oil was annually diſpenſed amongſt the ſick in our hoſpital, to the amount of fifty or ſixty gallons. The taſte and ſmell are extremely nauſeous; and it leaves upon the palate a ſavour like that of putrid fiſh. This remedy is moſt ſalutary when it operates by perſpiration; and the ſweat of thoſe to whom it is adminiſtered always becomes ſtrongly tainted with it. An oil of the ſame kind forms no inconſiderable part of the food of many northern nations; and it is ſaid to penetrate and imbue the deepeſt receſſes of the body ‡.

In

* See Hewſon on the Blood, p. 146.

† See Ruſſel's Hiſtory of Aleppo. X

‡ Oil was formerly adminiſtered in pregnancy, by Sir David

In the Philoſophical Tranſactions for 1750, (Vol. I. part ii. p. 295) Dr. Wright relates an experiment to prove that chalybeates do not enter the blood. He forced a dog, which had faſted thirty-ſix hours, to ſwallow a pound of bread and milk, with which an ounce and a half of green vitriol were mixed. An hour afterwards he opened the dog, and collected from the thoracic duct near half an ounce of chyle, which aſſumed no change of colour when the tincture of galls was dropped into it, though it acquired a deep purple from the ſame tincture when one fourth of a grain of ſal martis had been diſſolved in it. This experiment is uſually deemed deciſive in ſupport of the theory, that chalybeates exert their operations ſolely on the ſtomach, and that the vigour they communicate to the ſyſtem ariſes excluſively from their tonic powers on the alimentary canal, and on the ſympathy of the ſtomach with various other parts of the body. I am not inclined to doubt either the tonic action or the ſympathy ſuppoſed; but I ſee not that they preclude the imvid Hamilton, and other experienced phyſicians, to promote eaſy delivery: but modern theory has ſuperſeded their obſervation and experience.

mediate mediate agency of ſteel on remote parts of the human frame; for this remedy, in other forms capable of being introduced into the circulation, may exert conſiderable energy, as deobſtruent, ſtimulant, or aſtringent: and the experiment adduced only evinces that it did not ſubſiſt in the chyle as a vitriol qualified to ſtrike a black colour with galls. Neither does the calx of iron nor the glaſs of iron poſſeſs this power; yet, though changed, they are both capable of being reſtored to it. Perhaps, with equal reaſon, it might be preſumed, by one ignorant of chemiſtry, that ſal martis contains no iron, becauſe it is not acted upon by the loadſtone.

With the foregoing obſervation of Dr. Wright I ſhall contraſt thoſe made by the celebrated Dr. Muſgrave, which are alſo recorded in the annals of the Royal Society: — "I injected," ſays he, "into the jejunum of a dog, that had, "for a day before, but little meat, about "twelve ounces of a ſolution of indigo in "fountain water, and after three hours, open-"ing the dog a ſecond time, I obſerved ſeveral "of the lacteals of a bluiſh colour, which, on "ſtretching the meſentery, did ſeveral times "diſappear, but was moſt eaſily diſcerned when "the meſentery lay looſe — an argument that "the

" the bluiſh liquor was not properly of the

" veſſels, but of the liquors contained in it.

" A few days after this, repeating the experi-

" ment in another company, with the ſolution

" of ſtone blue in fountain water, and on a

" dog that had been kept faſting thirty-ſix

" hours, I ſaw ſeveral of the lacteals become

" of a perfect blue colour within very few mi-

" nutes after the injection; for they appeared

" before I could ſew up the gut.

" About the beginning of March following,

" having kept a ſpaniel faſting thirty-ſix hours,

" and then ſyringing a pint of deep decoction

" of ſtone blue with common water into one of

" the ſmall guts, and after three hours open-

" ing the dog again, I ſaw many of the lac-

" teals of a deep blue colour; ſeveral of them

" were cut, and afforded a blue liquor, ſome

" of the decoction running forth on the me-

" ſentery. After this I examined the ductus

" thoracicus, (on which, together with other

" veſſels near it, I had, on my return, made a

" ligature) and ſaw the receptaculum chyli and

" that ductus of a bluiſh colour; not ſo blue

" indeed as the lacteals, from the ſolution

" mixing, in or near the receptaculum, with

" lympha, but much bluer than the ductus

" uſed

" uſed to be, or than the lymphatics under the
" liver, with which I compared it, were *."

Stone blue is a preparation of cobalt, pot-aſh, and white lead, which, being converted into glaſs, is ground into a fine powder; and if ſuch a ſubſtance can pervade the lacteals, we may conclude that they are permeable to other bodies beſides thoſe deſigned for nutrition, and capable of aſſimilation with the blood. This argument, from analogy, receives great additional force from the known fact that mercury, and various other active remedies, may be conveyed into the body through the abſorbents of the ſkin, a ſyſtem of veſſels ſimilar to thoſe above mentioned in their ſtructure, uſes, and termination. In a caſe of hydrocephalus internus, on which I have lately been conſulted, a child under one year of age received, by ſucceſſive frictions, four ounces, ſix drachms, and two ſcruples of the unguentum cœruleum fortius between the 8th of February and the 7th of April, 1786. One ſcruple was adminiſtered each time; the operation took up more than half an hour; and the part to which the oint-

* See Philoſophical Tranſactions abridged by Motte, chap. iv. part ii. p. 76.

ment was applied was always previoufly bathed with warm water—precautions which feemed to fecure the full abforption of the mercury *. I fhould not omit to mention that the child recovered without any fymptoms of falivation, and continues perfectly well. Indeed I have repeatedly obferved that very large quantities of the unguentum cœruleum may be ufed in infancy and childhood without affecting the gums, notwithftanding the predifpofition to a flux of faliva at a period of life incident to dentition. The practicability of curing the hydrocephalus internus is a late and happy difcovery; and perhaps the peculiar efficacy of quickfilver in this alarming difeafe may reflect fome light on the general rationale of its action. The ftructure of the brain is yet very imperfectly known; but anatomifts have fufficiently afcertained that it is moft copioufly fup-

* Thirty-feven grains of calomel were given internally during this fpace of time, at proper intervals, and in fixteen dofes. The cafe referred to occurred in London, and was under the immediate direction of feveral phyficians of eminence. The ufe of mercury was adopted by my advice; and the eftimate of the quantity confumed, as made by the apothecary, has been tranfmitted to me by G. H. Efq. the father of the child.

plied with veſſels of every order, ſo that about one tenth of the whole maſs of blood circulates within it, although the weight of the encephalon does not exceed one fortieth part of the whole body *. On this large ſyſtem of veſſels mercury may be preſumed to act with a force proportionate to its magnitude and extent; and, in the inſtances when no ſalivation takes place, it is not unuſual for profuſe ſweatings to occur about the head. An acceleration of growth alſo, to an extraordinary degree, is frequently obſerved after the diſeaſe has been thus ſubdued. In one caſe, which fell under my direction in 1784, a young lady, nine or ten years of age, of a noble family in this county, increaſed in ſtature two inches within the ſpace of four months ſucceeding her recovery.

Whence is it that a medicine, ſo irritating as mercury, can be conveyed into the courſe of circulation, when even milk, or the mildeſt liquors, if transfuſed into the blood veſſels, have been found to produce convulſions and death? Is it that what paſſes by the lymphatics or lacteals is carried into the thoracic duct, and there

* See Monro on the Structure of the nervous Syſtem, p. 3, folio.

mixed mixed with a large portion of the chyle and lymph, by which its acrimony is ſheathed and diluted, or its chemical properties changed, before it enters the maſs of blood? For the abſorbents of the ſkin, and of the inteſtines, ſhould ſeem to require a capacity to bear the ſtimulus of thoſe extraneous bodies to which, in both ſituations, they are expoſed.

III. Medicines introduced into the courſe of circulation may affect the general conſtitution of the fluids, produce changes in their particular qualities, ſuperadd new ones, or counteract the morbific matter with which they may be occaſionally charged. By obſervations on the hæmorrhages, which have been ſuſtained without deſtruction to life; from experiments made on animals, by drawing forth all their blood, and by a computation of the bulk of the arteries and veins *; the maſs of circulating fluids has been eſtimated at fifty pounds in a middle-ſized man, of which twenty-eight pounds are ſuppoſed to be red blood. Fluids, bearing ſo large a proportion to the weight of the whole body, have aſſuredly very important offices in the animal œconomy. Endued with the com-

* Vide Halleri Prim. Lin. ſect. cxlix.

mon properties of other fluids, they are ſubject to mechanical laws; being variouſly compounded, they are incident to chemical changes; and as they are contained in a living vaſcular ſyſtem, their motions become ſubject to the influence of nervous energy.

But the proſecution of this ſubject will exceed the bounds of the preſent evening's diſcuſſion: and I ſhall reſerve what I have farther to advance upon it to ſome future meeting of the Society.

